# Mind-mindedness and styles of interaction of young fathers with their infants at three months: a pilot study

**DOI:** 10.1186/s40359-023-01480-0

**Published:** 2023-12-06

**Authors:** Elena Ierardi, Simona Fantoni, Margherita Moioli, Alessandro Albizzati, Cristina Riva Crugnola

**Affiliations:** 1 Department of Psychology, University of Milano-Bicocca, Milan, Italy; 2 Child Neuropsychiatric Unit 2, ASST Santi Paolo and Carlo, Milan, Italy

**Keywords:** Adolescent and young father, Father-infant interaction, Paternal mind-mindedness, Paternal sensitivity

## Abstract

**Background:**

Fatherhood at a young age can be characterized by a multiproblematic background with several risk factors that can negatively affect father-child relationships, the father’s well-being and child’s social-emotional development.

**Methods:**

This pilot study evaluated paternal interaction styles and mentalization in a sample of 22 young fathers and their 3-month-old infants and compared these variables with those of 22 adolescent and young mothers (the fathers’ partners). Parent-infant interaction were codified with Care-Index to evaluate styles of interaction and with Mind-Mindedness system to evaluate mentalization.

**Results:**

The results showed that young fathers had high scores in controlling behaviors and low scores in sensitivity, placing them in a risk range. The young father’s interaction profile did not differ from the young mother’s interaction profile. Infants had high scores in passive behaviors and low scores in cooperative behaviors, placing them in a high-risk range. Moreover, young fathers had more nonattuned mind-related comments than their partners.

**Conclusions:**

The findings indicate that low responsiveness and low mind-mindedness characterize the quality of adolescent and young father-infant interactions, highlighting the value of providing early intervention to support the father–child relationship, enhancing the father’s sensitivity and his ability to keep the infant in mind.

## Background

Fatherhood in adolescence and at a young age involves several psychological and social changes that may cause stress and conflict between parenting and teenager roles, with the consequent inability to integrate the developmental tasks typical of adolescence with those of fatherhood [1, 2]. Studies on adolescent fatherhood are scarce, unlike studies on adolescent motherhood. Indeed, the role of adolescent and young fathers has rarely been investigated because, in most cases, these young fathers do not live with their children [3]. However, the father’s presence or absence affects both the mother’s life and the child’s growth [4]. A small number of studies have shown that adolescent and young fathers are usually a few years older than adolescent mothers [5], and their main characteristics are similar to those of adolescent and young mothers in terms of risk factors [6]. In particular, in most cases, young fathers come from disadvantaged socioeconomic backgrounds, exhibit premature sexual risk behaviors, and have adverse childhood experiences; frequently, young fathers also have lower educational levels and a low chance of finding employment [7]. Furthermore, young fathers frequently experience substantial psychological, emotional, and social difficulties; for example, young fathers are twice as likely to be unemployed and to receive support allowance, frequently engage in criminal conduct, and demonstrate violent and hostile behaviors with alcohol and drug abuse that often persists after the birth of the child [8, 9]. One study [10] revealed that the adversities associated with parenthood at an early age made it harder for young individuals to break from the cycle of poverty within their birth families. Given that young fathers have lower educational levels and fewer economic opportunities than adult fathers, they have fewer opportunities to improve their social and economic conditions. In addition, compared to young mothers and adult parents, young fathers have more limited access to social services and local resources that can support their parental needs; thus, they may experience intense distress that increases the difficulties associated with the transition to a parental role, causing psychological and physical problems [11].

Sipsma and collegueas [10] also highlighted the possibility of an intergenerational cycle of adolescent fatherhood, similar to that of adolescent motherhood; i.e., the children of adolescent fathers may also become young parents. Low socioeconomic status, low educational level and high criminality risk, and inadequate parental support were identified as risk factors for sexual risk behaviors and an increased probability of becoming a father in adolescence. Finally, teen fathers are often portrayed as self-focused and destined to leave the mother and their children with little concern for their well-being [12, 13].

Regarding the consequences of adolescent and young fatherhood, various studies [6] have underlined that early fatherhood might cause a lot of difficulties, especially for those who grow up in multiproblematic families and communities. Indeed, young fathers are especially vulnerable to adverse mental health outcomes, economic issues, and unemployment [11] which in turn are risk factors for parental well-being [14, 15]. Lee and colleagues [16] found that the risk for developing depressive symptoms is twice as high in adolescent fathers as it is in adult fathers; adverse experiences, parental stress, lack of social support, and interpersonal conflicts might also increase the risk of developing or reactivating depressive symptoms in young fathers [17].

The only available review from the last 20 years about fatherhood in adolescence confirms several negative consequences of becoming a father at a young age, such as several mental health issues, substance abuse, a low educational level, poverty and unemployment, insufficient parental involvement, and poor parental satisfaction [8].

### Young father-infant interaction

Literature on the quality of father-child interaction shows that the paternal style of interaction has characteristics that differentiate it from the maternal style of interaction [18, 19], as it is more focused on physical play, such as tickling, “*rough and tumble play*”, chasing and bouncing on the knee. Zeegers and colleagues [20] defined the paternal style of interaction as lively, excitatory, unpredictable and emotionally arousing and speculated that this style has a specific effect on child development and mental health. Furthermore, fathers seemed more directive than mothers in their interactions with their children, using more restrictions and limits [21]. Moreover, Tamis-LeMonda et al. [22] observed that fathers and mothers do not differ in their total number of utterances, the length of the utterances or word types used during interactions with their children. However, fathers frequently use more directive language focused on the activities, while mothers more often repeat the children’s expressions to better understand what the child is saying or doing.

Moreover, fathers with no occupation and a lower educational level than fathers with an occupation and a higher educational level had less positive styles of interaction with their children [23].

Research on father–child interaction in adolescent and young fathers is scarce. To our knowledge, the only study [24] comparing adolescent parent–infant interaction showed that young fathers are less sensitive and less engaged in social play than young mothers. In this regard, the authors suppose that mothers might receive more education and support related to parenthood and childcare from healthcare and educational practitioners than fathers.

An important issue that could affect the quality of father–child interaction is the relationship between the father and mother. Easterbrooks and colleagues [25] studied a sample of adolescent and young fathers and observed that a nonconflictual, supportive and deep relationship with the partner was associated with a greater involvement of the youth in the family and with a greater use of positive parenting strategies (reflecting and paying attention to the child’s needs) instead of physical punishment and threats. The positive impact of adolescent fathers was also seen in the socioemotional development of the children [26]: children with fathers with good parenting skills and who financially contributed to family wealth had better cognitive and linguistic development [27], were less likely to be neglected [28] and had fewer behavior disorders and more secure attachment bonding [29]. In contrast, fathers who are absent or display antisocial behaviors might negatively affect the child’s development, leading to a higher risk of behavioral problems [30]. No studies have examined the mentalization ability of young fathers, which has been studied in adolescent mothers; young mothers show poorer mind-mindedness and reflective functioning than adult mothers [31–34]. Parental mentalization is the parent’s ability to understand behavior (his or her own as a parent and that of their child) and is based on their underlying mental states [35]; parental mentalization is considered crucial within parent–child relationships [36]. Limited studies have examined mentalization in adult fathers, and the results have been mixed [37–39]. One study showed that the mentalization of mothers and fathers when interacting with their children is independent and that mothers showed slightly higher levels of mentalizing than fathers [40]. Another study [41] demonstrated no difference between mothers and fathers in terms of the number of mind-related comments; however, fathers contributed more comments about problem-solving and fewer ‘speaking for the babies’ than mothers.

### Approach of our study

As only one study has evaluated adolescent father–child interactions, our pilot study may fill a gap in the literature on the quality of interaction styles and the mentalizing ability of young fathers. This quality may affect the child’s development independently from the other parent or may compensate or reinforce the impact of the other parent’s sensitivity and mind-mindedness. The general purpose of our pilot study with three exploratory aims is to examine the quality of young father–child interaction styles and fathers’ mentalization with infants at three months of age.

The first aim was to assess the quality of the styles of interaction and paternal mind-mindedness in adolescent and young father–infant interaction. The second aim was to identify whether some social and demographic variables, such as the father’s age or employment, influence the father’s quality and style of interaction and mind-mindedness. The third aim was to compare young fathers’ style of interaction and mind-mindedness level with that of their respective partners (young mothers’).

## Method

### Participants

Adolescent and young parents and their infants were recruited from the “Accompagnamento alla genitorialità in adolescenza” [Accompanying Parenting in Adolescence] Service at the ASST Santi Paolo and Carlo Hospital of Milan, a service that follows adolescent and young mothers aged 21 years and below and their partners during the perinatal period.

The inclusion criteria for participation in the study were being a father partner up to 24 years old and a partner of a mother under the age of 22 (see the sample of Easterbrooks et al. [25] who recruited young fathers aged 15 to 24 years); an ability of speaking and understanding the Italian language; the presence of a partner; an uneventful delivery; infants born at full term with no medical complications and being physically healthy. Those with twins or premature babies were excluded.

Parents and their infants were contacted when the infants were 2 months old. Parents interested in participating in the research were re-contacted and evaluated when the infants were 3 months old.

The sample consists of 44 adolescent and young parents and their infants; therefore, 22 pairs of parents took part in the research. Female infants accounted for 50%. Fathers were between 16 and 24 years old (M=19.68; SD=2.16), 40% was adolescent fathers (16–19 years old) and 60% was young fathers. They had an educational level between 8 and 13 years, and 73.7% were employed. Mothers were between 13 and 21 years old (M=17.32; SD=2.03), had an educational level between 8 and 13 years (M=11.14; SD=2), and 91% were unemployed. 80% of the parents had a low socioeconomic level, and 20% had a medium socioeconomic level.

A total of 72.2% of the fathers and 81.8% of the mothers were Italian. 54% of the couples lived together. All the fathers had a couple relationship with the mother. The remaining parents were European or Latin American, knew the Italian language and were integrated into the Italian cultural context. Finally, 94.7% of the couples stated that the pregnancy was not desired. The study protocol was approved by the institutional review board of the ASST Santi Paolo and Carlo Hospital of Milan. All subjects gave their written informed consent.

### Procedure

When the infants were 3 months of age, the parent–infant dyads were video-recorded in a hospital playroom containing a small mattress, where the parent and infant could sit or lie, and a few age-appropriate toys for the baby.

The parents were instructed to interact with the infant as they would at home for 5 min, according to the manual of the Child Adult Relationship Experimental Index (Care-Index) [42] and the manual of Mind-Mindedness coding system [43] that indicated that 3–5 min in the field of early interaction are sufficient to evaluate both respectively styles of interaction and mind-mindedness. The dyadic interactions were coded with the Care-Index [42] to evaluate parental and infant style of interaction and behaviors and with the Mind-Mindedness coding system [43] to evaluate the parental mentalizing abilities.

### Measures

#### Parent-infant styles of interaction

Interactions were video-recorded and evaluated with the Care-Index [42], a method that codes interactions based on 7 behavioral characteristics: facial expressions, vocal expressions, body position and contact, affection, turn-taking, control, and choice of activity. Parental styles of interaction are assessed on three scales: Sensitivity with responsiveness toward the emotions and actions of the child; Controlling with hostility and intrusiveness toward the activities of the child; and Unresponsive with physical and emotional detachment. The styles of interaction of the child are assessed on four scales: Cooperative with expression of positive emotions and acceptance of actions undertaken by the parent; Compulsive-compliance with cautious and inhibited behavior and a compliant approach toward the parent; Difficulty with resistance to proposals of the parent; and Passive with physical and emotional withdrawal.

The scores vary from 0 to 14 across the scales. Regarding the parental sensitivity scores, 0–4 is considered high risk, 5–6 indicates marginally adequate parental sensitivity, 7–10 indicates adequate sensitivity, and 11–14 indicates very good sensitivity.

The Care-Index is a method for evaluating the quality of adult-infant interaction. Although the adult is most often the mother, the procedure can be used with fathers [42].

Reliability between observers was calculated for 20% of the dyad observations through the intraclass correlation coefficient and was ICC=0.82 for paternal and maternal behavior and ICC=0.74 for infant behavior. Reliability was also calculated with Cohen’s Kappa [44] and it was K=0.80 for paternal behaviors, K=0.81 for maternal behaviors, and K=0.78 for infant behaviors.

#### Mind-mindedness

Parental mind-mindedness was assessed from a video-recorded 5-minute free-play session between parent and child, using the Mind-Mindedness coding system [40]. This procedure can be used with mothers and fathers [43, 45].The father’s and mother’s speech during the sessions was transcribed verbatim. The comments were divided into comments unrelated to the infant’s mind or emotion (not mind-related) and comments that included an internal-state term related to the infant’s mind or emotion (mind-related comments). Mind-related comments included references to wishes and desires, mental states, mental processes, emotions, attempts to manipulate people’s beliefs and comments where the mother “put words into her infant’s mouth”. A mind-related comment was also classified as an appropriate mind-related comment if one or more of the following conditions were met: (a) the independent coder agreed with the mother’s reading of her infant’s internal state, (b) the internal state comment linked the infant’s current activity to similar events in the past or future, (c) the internal state comment served to clarify how to proceed if there was a lull in the interaction, or (d) the parent voiced (using the first person) what the infant might say if he or she could speak.

The mind-mindedness score represented the number of mental descriptors expressed as a proportion of the total number of descriptors used to control differences in parental verbosity. Higher proportional scores indicated greater mind-mindedness. Interrater reliability was K=0.92 for paternal mind-related comments, K=0.90 for paternal appropriate mind-related comments, K=0.92 for maternal mind-related comments, and K=0.92 for maternal appropriate mind-related comments.

### Data analysis

The SPSS Statistic 27 package was used for all analyses. Preliminary analyses with t tests did not show significant sex differences regarding parental and infant styles of interaction or mother and father mind-mindedness. First, descriptive statistics on father–infant interaction styles and paternal mind-mindedness were developed to outline the quality of the interaction. Correlation analyses were used to examine the associations between paternal and infant styles of interaction and the father’s mind-mindedness at the exploratory level. Multiple regression analyses were used to evaluate the effect of risk factors (father’s age and occupation) on father–infant interaction styles and paternal mind-mindedness. Finally, to investigate potential differences between the young fathers’ profiles and their partners’ profiles on an exploratory level, paired sample t tests were used to compare paternal and maternal Care-Index styles and paternal and maternal mind-mindedness.

## Results

### Styles of interaction and mind-mindedness of adolescent and young father-infant dyads

Regarding styles of interaction of adolescent and young fathers with their infants, analyses at a descriptive level based on Care-Index categories showed a score of M=6.19 (SD=2.76) in the sensitive style, which was in the at-risk range regarding relationship quality according to Crittenden (1998). This risk range is distinguished by unsolved problems and restricted playfulness of parent, and it is considered to be in need of “future intervention”. In addition, the controlling style score was high (M=6.43, SD=2.89). This score is considered high as it corresponds to almost half of the total points to be awarded. Whereas the unresponsive style score was low (M=1.19, SD=1.32).

Concerning the infant style of interaction with fathers, the cooperative style score was low (M=4.14, SD=3.10), representing a high risk for inadequate relationships. This risk range is distinguished by missing playfulness and scarce dyadic attunement, as indicated by Crittenden, who suggests parent‒child psychotherapy as an intervention. On the other side the passive style prevailed, with a score of M=6.62 (SD=2.88) (see Table 1). This score is considered high as it corresponds to almost half of the total points to be awarded.

**Table 1:** Comparison between young father’s and young mother’s measures of style of interaction and Mind-Mindedness

	Fathers		Mothers			
	*M*	*SD*	*M*	*SD*	*t*	*p*
Sensitivity	6.19	2.76	6.90	3.12	0.78	0.44
Controlling	6.43	2.89	5.38	3.15	-1.18	0.24
Unresponsive	1.19	1.32	1.81	2.67	1.08	0.29
Cooperative	4.14	2.97	5.52	2.97	1.65	0.11
Compulsive-Compliance	1.57	1.85	0.38	1.32	-3.62	0.002**
Difficulty	1.62	1.98	2.38	3.91	1.08	0.29
Passive	6.62	2.88	5.76	3.50	-0.92	0.36
Mind-related total	0.08	0.10	0.02	0.02	-2.48	0.023*
Not mind-related total	0.14	0.13	0.11	0.08	-0.72	0.47
MM appropriate	0.03	0.04	0.01	0.02	-1.73	0.10
MM nonattuned	0.06	0.07	0.01	0.01	-2.39	0.033*

***Note***: *mean (M), standard deviation (SD), Student’s t-test (t), significance level (p)*

*p<0.05; **p<0.01

Regarding the assessment of paternal mind-mindedness, at a descriptive level, mind-related comments scored M=0.08 (SD=0.10), and not mind–related comments scored M=0.14 (SD=0.13); the appropriate mind-related comments score was M=0.03 (SD=0.04), whereas the nonattuned comments score was M=0.06 (SD=0.07). Adolescent and young fathers’ provided appropriate comments less frequently than the non-risk sample of adult fathers (M=0.08, SD=0.06) [20], and young fathers more frequently contributed nonattuned comments, than those of the same non-risk sample of adult fathers (M=0.01, SD=0.02) [18] (see Table 1).

Furthermore, associations between paternal styles of interaction and mind-mindedness scores were investigated. The Pearson’s R correlations did not show significant associations between Care-Index styles and mind-related comments (see Table 2).

**Table 2:** Correlations between father’s styles of interaction and Mind-Mindedness

	MM appropriate	MM nonattuned
Sensitivity	− 0.14	− 0.34
Controlling	0.30	0.09
Unresponsive	− 0.33	0.00

### Effects of sociodemographic variables

Sociodemographic variables were examined, i.e., the father’s age and employment status, to assess the potential effects of these variables on the styles of interaction and mind-mindedness. Therefore, the regression analysis used paternal styles of interaction and paternal mind-mindedness scores as dependent variables and paternal age and employment/unemployment as independent variables.

We conducted separate regressions for each interactive style and mind-mindedness scores as dependent variables.

First, we used father’s age as an independent variable. The analysis revealed no significant predictive effects related to the father’s age. In particular, the father’s age was not predictive of the sensitivity (B=0.11, t=0.38; *p*=0.70), controlling (B=-0.15, t=-0.50; *p*=0.61), or unresponsive (B=-0.02, t=-1.26; *p*=0.90) styles or of appropriate mind-related (B=0.00, *t*=-0.72; *p*=0.47) and nonattuned mind-related (B=0.00, t=1.00; *p*=0.32) comments. Second, we tested paternal employment/unemployment as an independent variable. A significant effect was found for the paternal employment/unemployment. Being employed was predictive of a more sensitive style (B=4.02, t=3.42; *p*=0.003) and, trending toward significance, a less controlling style (B=-2.77, t=-1.96; *p*=0.06); in contrast, being employed was not predictive of an unresponsive style (B=-1.05, t=-1.01; *p*=0.32) or of appropriate mind-related (B=-0.23, t=-1.04; *p*=0.32) or nonattuned mind-related (B=-0.02, t=-0.09; *p*=0.92) comments.

### Paternal and maternal risk profiles

Paired sample t tests were used to examine paternal and maternal interaction styles and mind-mindedness to compare adolescent and young fathers’ risk profiles with their respective partners (see Table 1).

The results showed only one significant difference in interaction styles — the infant compulsive-compliance style. Infants had higher compulsive-compliance behaviors with fathers than with mothers. No significant differences were found regarding the other parental and infant interaction styles. Moreover, a poor sensitivity style was observed in the interaction of both parents with infants, with scores in the risk range due to parental low sensitivity and high intrusiveness. Infants showed poor cooperative styles, placing them in the risk range for interaction with mothers and in the high-risk range for interaction with fathers [39].

Finally, young fathers made more nonattuned mind-related comments than mothers. In addition, appropriate mind-related comments were less frequent in both young fathers and mothers than in a sample of adult parents. A post hoc power analysis indicated that a sample of 44 participants was sufficient to detect a medium effect size with a power of 0.90 (α=0.05).

## Discussion

This is the only recent study to examine the quality of the interaction styles between adolescent and young fathers and their infants, and is the first to assess the ability of mentalization in young fathers. The results of our pilot study showed that adolescent and young fathers had low sensitivity and high controlling style scores, displaying intrusive and aggressive behaviors when interacting with their 3-month-old infants. These scores placed them in a risk range for sensitivity, according to Crittenden’s model [42]; parenting support intervention is suggested for scores in this range. Infants had, in turn, more passive, compulsive-compliant and less responsive behaviors when interacting with fathers, placing them in a high-risk range that, according to Crittenden’s model, requires parent‒child psychotherapy. Interactive exchanges within the dyad of an adolescent father and his 3-month-old infant were characterized by scarce positive emotions, with a predominance of negative affectivity, poor attunement, and little shared play. With regard to sensitivity, this interaction profile had similar features to the one found by McGovern [24].

Moreover, the quality of paternal styles of interaction was similar to that of their respective adolescent partners. The analyses revealed no significant differences between fathers and mothers, as both paternal and maternal low sensitivity and high intrusiveness scores were in the risk range for parenting quality and indicated a need for support interventions [42]. Regarding young fathers, we found that they had a multiproblematic life circumstances as low socio-economic, low level of education, and absence of job may be risk factors for parenting, similar to those of adolescent and young mothers [32].

Another interesting result concerned the differences between the infant styles of interaction with the mother and father. Infants had higher scores in the compulsive-compliance style when interacting with their adolescent and young fathers than when interacting with their mothers. According to Crittenden [42], a child’s compulsive-compliance interaction style is characterized by circumspect and inhibited behaviors that allow the infant to interact with a parent in a compliant and indirect way, with mechanical gestures and without emotional expression to the parental proposed activities. This kind of interaction is often observed in children who experience abuse or neglect during childhood [46]: to deal with these adverse experiences, they use coping strategies that reduce the parent’s inclination to commit further abuse and then adopt compliant behaviors that could increase the chance of sharing enjoyable interactions with their father or mother. In the short term, this strategy may be adaptive for the neglected or abused child; however, in the long term, it may become an obstacle to the construction of the child’s skills and to the development of his or her identity, as the infant’s real feelings are falsified to cope with a complex and painful reality.

An original result of our pilot study that never emerged in previous research regards the mind-mindedness ability of young fathers. The low frequency of appropriate mind-related comments and the high frequency of non mind–related comments and nonattuned mind-related comments found in young fathers highlighted their difficulty in understanding, attuning to and verbalizing children’s intentions, desires, thoughts and feelings, showing a nonattuned attribution of states of mind to the child. Similarly, studies that compared adolescent and adult mothers found that younger mothers made fewer mind-related and appropriate mind-related comments, more nonattuned mind-related comments and fewer comments with positive emotions than adult mothers [31, 33].

Young fathers’ mind-mindedness was also found to be lower and more at risk in terms of nonattuned mind-related comments than that of adolescent mothers. These results agree with Cooke’s research on adult parents, according to which mothers had slightly higher mentalizing levels than fathers [40]. In any case, young fathers and young mothers showed poor mind-mindedness with a low frequency of appropriate mind-related comments and more frequent non mind–related and nonattuned mind-related comments.

The correlation analyses showed no significant relationship between interaction styles and mind-mindedness in young fathers. This result agrees with some previous studies regarding the lack of association between the quality of styles of interaction and mind-mindedness in samples of mothers [47, 48]. It can be assumed that these findings represent different aspects of the quality of interaction, as interaction styles outline the profile of parental sensitivity and affective attunement, whereas mind-mindedness outlines representational aspects of parental mentalizing.

Another significant result concerned paternal unemployment, which was predictive of a less sensitivity style of interaction and, at a level of tendency towards significance, a more controlling style. Indeed, the literature indicated that a low family SES, an economic and social combined total measure of social, economic, and working status of individuals, was a risk factor for the well-being of the parents and the development of the infant and their relationship [14, 15]. Furthermore, Futris et al. [23] showed that fathers with an occupation and a higher educational level were more willing to interact and had a more positive relationship with their children.

Paternal age was not predictive of style of interaction and mind-mindedness. A larger sample of young fathers could reveal any differences related to the father’s age in more detail by dividing them into very young (16–17 years) and young (18–23 years) groups and evaluating risk and protective factors that may influence the quality of paternal interaction and mentalizing more deeply.

### Limitations

First, the sample size was not large, mainly due to the complexity of reaching this unique population of adolescent and young parents. Additionally, the frequency of young fathers not living with their partners and their children made their involvement very difficult. Another limitation concerns the assessment of parent‒infant interaction, which took place only when the infant was three months of age. Moreover, Care-Index and MM coding system were more often used to evaluate mother-child interactions, although in recent years they have also been applied to evaluate father-child interactions and paternal mentalization. Further longitudinal research may be useful in examining the trend and evolution of parental styles of interaction quality and mind-mindedness during the child’s development.

In addition, other variables that could influence paternal parenting were not considered, such as psychopathological problems, mental health, and the social or criminal environment in which the family lives. A further limitation concerns not considering infant temperament and its possible effect on parent–infant interaction.

## Conclusions and clinical implications

Parent‒child relationships have been studied for many years, starting from the investigation of the bond that the child establishes with the mother, a figure considered the main source of care and attention. Only recently has the focus shifted to the role of the father, allowing us to analyze the characteristics, peculiarities, strengths, and limitations of paternal parenting. Interest in the paternal figure has allowed an increase in research into fathers at risk during parenting, such as fathers experiencing depression or other mental disorders, fathers who use drugs, who are in prison, and who are in adolescence or at a young age, observing the quality and characteristics of their interaction with their children.

In this regard, our pilot study deepens our understanding of the quality of the interactions of young fathers with their infants in the first months of the infant’s life, highlighting that the interaction of young father-infant dyads was characterized by paternal low sensitivity, high intrusiveness and poor mind-mindedness, and more compulsive-compliant and less sensitive infant behaviors. This high-risk profile was even more negative than that of adolescent and young mother–infant dyads.

The results of our pilot study should be considered preliminary, given the low sample size. However, the study provides some initial guidance for implementing specific intervention programs aimed toward both adolescent mothers and the fathers of their infants to support them in their involvement with their babies, focusing on improving their sensitivity and mentalization skills. In this regard, Barr et al. [49] implemented “The Baby Elmo Program” to support the parental education of incarcerated teen fathers with children between 1 and 15 months old to improve the quality of the father–child interaction. The intervention offers a combined proposal of training based on the use of media and educational videos, which introduce themes such as attachment, childhood exploration tuning with child’s requests, and active father–child interaction. The results showed improved verbal and nonverbal communication, a higher frequency of secure attachment bonding and more positive relationships between the father and their child during the imprisonment period, leading to developmental benefits for both the child and the adolescent father. Another example is the PRERAYMI intervention aimed at young mothers [50, 51], which effectively increases maternal sensitivity and mind-mindedness and could be extended to fathers.

## Data Availability

The data that support the findings of this study, as well as the used materials, are available from the corresponding author upon reasonable request.
